# Usual interstitial pneumonia coexisted with nonspecific interstitial pneumonia, What’s the diagnosis?

**DOI:** 10.1186/1746-1596-7-167

**Published:** 2012-12-03

**Authors:** Xia Fang, Benfang Luo, Xianghua Yi, Yu Zeng, Fang Liu, Huiping Li, Pan Gu, Xuyou Zhu, Suxia Zhang, Gelin Jiang

**Affiliations:** 1 Department of Pathology, Tongji Hospital, Tongji University School of Medicine, Shanghai, 200065, China; 2 Department of Respiratory medicine, Shanghai Pulmonary Hospital, Tongji University School of Medicine, Shanghai, 200433, China; 3 Department of Special Examination, Shanghai Pulmonary Hospital, Tongji University School of Medicine, Shanghai, 200433, China

**Keywords:** Nonspecific interstitial pneumonia, Usual interstitial pneumonia, Idiopathic nonspecific interstitial pneumonia, Diagnosis

## Abstract

The differential diagnosis between idiopathic nonspecific interstitial pneumonia(INSIP) and idiopathic pulmonary fibrosis(IPF)/usual interstitial pneumonia(UIP)is tough in both clinicians and pathologists. In this study, we analyzed the lesions of right lung removed from a 58-year-old patient by gross and microscopy. The results showed that the pathological appearance of nonspecific interstitial pneumonia (NSIP) and UIP coexisted in his upper lobe. Besides, because of severe fibrosis in middle and lower lobes, it was hard to distinguish the lesions of NSIP fibrotic pattern (NSIP-F) or UIP. Based on clinic-radiologic-pathological data, the diagnosis of INSIP-F was made for this patient finally. Our study suggests that UIP is not always an accurate diagnosis when the NSIP and UIP coexist, and NSIP can have regions of UIP.

**Virtual slide:**

The virtual slide(s) for this article can be found here: http://www.diagnosticpathology.diagnomx.eu/vs/2573531681608730

## Background

Because of different treatment and prognosis for idiopathic nonspecific interstitial pneumonia(INSIP) and idiopathic pulmonary fibrosis (IPF) **/** usual interstitial pneumonia (UIP), accurate diagnosis for this two diseases become critical for clinicians and pathologists [1-3]. However, the differential diagnosis among them is hard for both clinicians and pathologists [4], especially between INSIP fibrotic pattern (INSIP-F) and IPF/UIP. Some experts often prone to make the diagnosis of UIP when the pathological appearance of UIP and NSIP exist at the same time [5]. Up to now, pathological descriptions for NSIP are mainly from biopsy specimens, lacking the observation of whole lung sample from operation, which results in unilateral understanding of such lesion. Here, we report one case of idiopathic interstitial pneumonia (IIP) diagnosed by clinic-radiologic-pathological (CRP) method, in which UIP pattern existed with NSIP; the sample was a whole right lung removed from pneumonectomy.

## Case presentation

**Figure 1:**
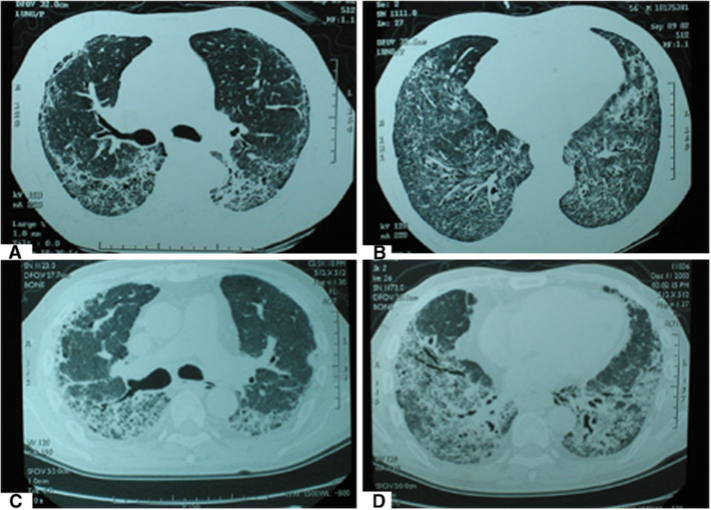
**The examination of radiology.** The chest CT displayed both lungs had reticular and ground glass opacifications, which located predominantly in subpleural and two lower lobes for two years before lung transplantation (**A**, **B**). To recheck CT after one year, above lesion had aggravated, showing fibrous strips with traction bronchiectasis (**C**, **D**).

Here’s a 58-year-old man with a history of smoking without dust and poisons contact, who came to Shanghai Pulmonary Hospital in July 2004 because of repeated cough, expectoration and progressive shortness of breath for three and a half years. Two years ago his chest CT showed that the lower lateral region of bilateral lung had reticular and ground glass opacifications without honeycomb (Figures 1A~B). Examination of pulmonary function showed restrictive ventilator and diffusion function disorder (FVC 72.3%, FEV_1_ 72.6%, VC 78%, TLC 75.8%, D_L_CO/V_A_ 78.3%). The blood gas analysis was regular (PH 7.4, PaO_2_ 96mmHg, SO_2_ 95%, PaCO_2_ 40mmHg). Besides, there was no specific lesion to be found through transbronchial lung biopsy, thus we made a diagnosis of IIP, based on clinical and radiographic information.

After treatment with glucocorticoid in the initial two years, his symptoms were reduced and pulmonary function was improved greatly, but the absorption of lesions wasn’t manifest. One year later, his chest CT displayed aggravated lesions, showing fibrous strips with traction bronchiectasis (Figures 1C~D). Pulmonary function disorder became more severe than that of two years ago (FVC 54.2%, FEV_1_52.7%, VC 67.9%, TLC 63.5%, D_L_CO/V_A_ 66.1%), accompanying anoxemia and type I respiratory failure (pH 7.4, PaO_2_ 58mmHg, PaCO_2_ 35 mmHg, SO_2_ 88%). With estimation for body condition, right lung transplantation was operated for this patient, but he died of respiratory failure after two weeks even if the surgery was successful.

**Figure 2:**
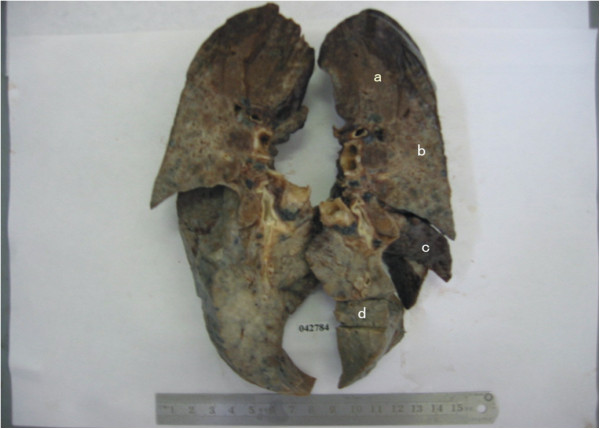
**The pathological gross examination of whole lung specimen.** Examining removed right lung, the apicoposterior segment of upper lobe was dark red, anterior segment was grey red alternated with grey white, middle and lower lobes was predominantly grey white. Getting specimenes from apicoposterior segment of upper lobe (a), anterior segment (b), middle (c) and lower lobe (d) respectively.

**Figure 3:**
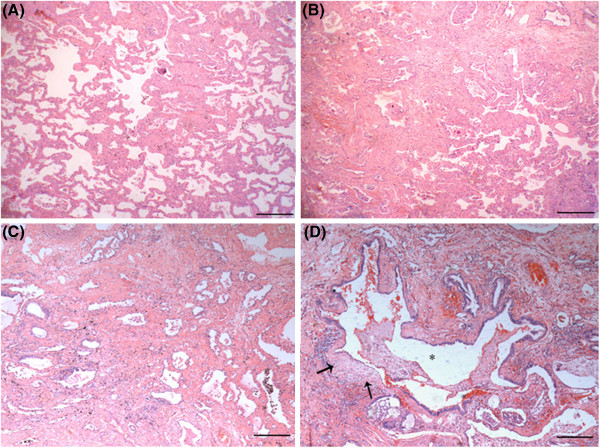
**Microscopic features of the patient's lung upper lobe.** Under low power microscope, some alveolar septum of apicoposterior segment of upper lobe(from where labeled “a” in Figure 2 ) had more lymphocyte and plasmocyte infiltrated, alveolar wall was mild broadening, but absent evident of fibrous hyperplasia which looks like pattern of NSIP cellular type (**A**); Some alveolar wall had fibre hyperblastosis obviously , a few collagen deposited and lymphocyte infiltrated as picture of NSIP mix type (**B**) and fibrotic type (**C**) ; The fiber interval of subpleural aera of anterior segment (from where labeled “b” in Figure 2 ) was thickening clearly, bronchiole epithelium had metaplasia and cystic fibrous gas cavity was formed (*), several cystic wall had small fibroblast foci (solid arrow), The appearance was extremely like UIP (**D**). ( H&E stain, Magnification:HE×40).

Subsequently, the removed right lung was performed on pathological examination. Gross observation demonstrated that the section was general consolidated and the lesion was mild in upper lobe but severe in middle and lower lobes (Figure 2). Three pieces of tissues were taken from each lobe (1.8×1.5×0.5 cm^3^ for each piece of tissue). Under light microscope, the apicoposterior segment of upper lobe was characterized by the pattern of NSIP, including expansion of the interstitium, a variable extent of chronic inflammation and fibrosis. In some regions, it showed mild hyperplasia of fiber tissue in alveolar septum and infiltration of many lymphocytes, similar to the pattern of Cellular NSIP (NSIP-C) (Figure 3A). Interestingly, in other regions, fibrotic NSIP (NSIP-F) was distinct, showing thickened alveolar septum with collagenous and dense fibrosis in nature (Figure 3B, C). It can also be found that NSIP-C and NSIP-F migrated with each other (Figure 3B). In the subpleural area of anterior segment of upper lobe, narrowed alveolar-space owing to the dense interstitial fibrosis and collagen deposit was present. The main features were an existence of a few small fibroblast foci in cystic fibrous gas cavity wall (so-called microscopic honeycomb lung) except bronchiole epithelium metaplasia, which are usual trait of UIP (Figure 3D).

**Figure 4:**
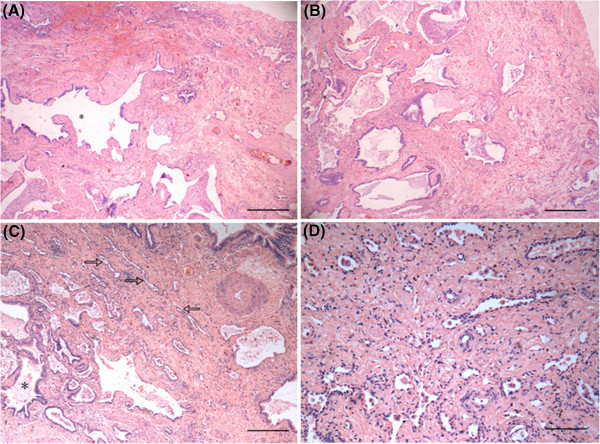
**Microscopic features of the patient's lung middle and low lobes.** Under low power microscope, the structure of middle (**A**,**B**) and lower lobes (**C**) were basically same (from where labeled “c and d” in Figure 2), The proliferative fibrous tissue thickened alveolar wall, resulted in the narrowness of alveolar space, even obstruction(hollow arrow). Some regions had fibrosis and consolidation, alveolar structural remodeling and many cystic fibrous gas cavity (*). NSIP-F or UIP was not easy to distinguish. In lower lobe, it is still visible the diffuse fiber hyperblastosis and residual alveolar structure in fibrotic tissues,indicating NSIP-F (**D**). ( H&E stain, Magnification:HE×40).

**Figure 5:**
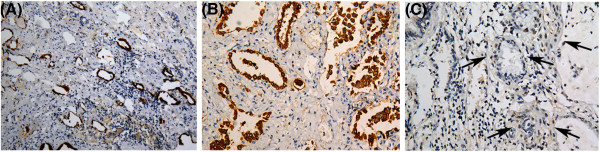
**A-C Immunohistochemical staining of cytokeratin(AE1/AE3), SP-A and P53 protein.** The tissue obtained from upper lobe of right lung. Cytokeratin was positive in residual alveolar epithelium of fibrosis tissue (**A**, EnVision × 100).The strongly positive expression of SP-A is observed in hyperplastic type II alveolar epithelium (**B**, EnVision×200), and some hyperplastic alveolar epithelium demonstrated positive expression of P53 (**C**, arrow. EnVision × 200).

Furthermore, the structures of middle (Figures 4A, B) and lower lobes (Figure 4C) were basically same and had similar histological changes with that of the subpleural area of anterior segment, demonstrating alveolar structural remodeling and fibrosis consolidation. Some region appeared the pattern of NSIP-F, while some had many cystic gas cavities with mucus embolus and alveolar structural remodeling, which made it difficult to distinguish NSIP-F from UIP. In addition, it was still visible the residual alveolar structure in the fibrotic consolidated area of low lobe (Figure 4D).The immunophenotype of residual alveolar epithelium in fibrosis tissue were consistently positive for cytokeratin(AE1/AE3) (Figure 5A) , with a strongly positive expression of Surfactant Protein-A (SP-A) in hyperplastic type II alveolar epithelium (Figure 5B). Weak expression of P53 in some hyperplastic alveolar epithelium can also be found (Figure 5C). Comprehensively, the patient was diagnosed as INSIP-F by CRP method.

## Discussion

American Thoracic Society(ATS) reported a comprehensive research about INSIP in 2008. Among the 193 cases of NSIP from published data, they found that only 67 cases were INSIP (34.72%), 28 cases of them were UIP (14.51%). Through which we can see it is difficult for clinicians and pathologists to distinguish INSIP from UIP. Histologicially, NSIP-F has a uniform pattern, characterized by expansion of the interstitium, a variable extent of chronic inflammation and fibrosis which can be collagenous or fibroblastic, lacking or scarcity of fibroblastic foci, primarily distinguishing it from UIP.

Our case showed various histological appearance of upper lobe. Not only was there the migration between NSIP-C and NSIP-F, but also the morphological appearance of NSIP and UIP coexisted in upper lobe. For example, formation of cystic fibrous gas cavity with fibroblast foci was hard to be differentiated from honeycomb, the latter and fibroblast foci were the morphological features of UIP. Besides, pathological appearance of middle and lower lobes showed the severe fibrosis and consolidation, formation of cystic gas cavity and alveolar structural remodeling and so on. Aforementioned pathological features render us hard to make a diagnosis of NSIP-F or UIP. Final diagnosis were based on the following points: (1) Without honeycomb change in chest CT scan, which conforming to NSIP-F; (2) Response to glucocorticoid to some extent; (3) Typical feature of NSIP in light fibrosis region; small fibroblast foci both in size and amount which occupied less than 10% of all slides, and looser collagen than myogelosis occurred in UIP. If the materials were all from only one lobe or the size of tissue was too small, the patient would possibly be misdiagnosed, or at least we would hardly make the diagnosis as NSIP-F or UIP.

How to make diagnosis when the histological pattern of NSIP coexists with UIP in multiple lobes biopsies? Flaherty et al. [5] found that 26% of 109 patients had both the pattern of UIP and NSIP after analyzing several lobes of lung biopsy, so they thought as long as one lobe had the appearance of UIP, these patients should be diagnosed as UIP because of the poor prognosis of it. Recently, ATS/European Respiratory Society (ERS)/Japanese Respiratory Society (JRS)/Latin America Thoracic Society (ALAT) established the diagnosis guideline of IPF which concluded that some puzzling cases of IPF should be diagnosed based on combination with pathological information and HRCT [6], and the appearance of honeycomb lung showed in HRCT is an important feature of UIP, whereas our case showed the pathological appearance of NSIP together with UIP, without honeycomb lung, so after a comprehensive analysis of all slides, we diagnosed our case as INSIP with UIP-like areas.

Differential diagnosis of INSIP includes drug induced lung injury [7], the terminal stage of eosinophilic pneumonia and lung damage caused by environment exposure [8]. However, our case had no usage of cytotoxic drugs and immunosuppressants, occupational and environmental exposure history. Thus it is easy to discriminate our case from above other diseases. Kirby et al. [7] reported that 9 out of 28 recipients with renal allograft history who had pulmonary complications, including pulmonary hemorrhage, organizing pneumonia and pulmonary alveolar proteinosis, but no NSIP was found. We also excluded eosinophilic pneumonia, because our patient didn’t show an increasing number of eosinophile granulocytes in his peripheral blood and eosinophile granulocytes infiltrated in his lung lesions.

Histologicially, cystic fibrous gas cavity can be found in the whole right lung in our case, and the lesion almost existed in terminal stage of chronic lung disease. We assume that the obstruction of proximal bronchial results in extension of distal bronchial gradually, which may be the cause of the lesion, but the molecule mechanisms of the lesion are elusive. Besides, the metaplasia of bronchial was also a predominant feature in our case, some region even had hyperplasia of alveolar epithelium and abnormal expression of P53 (Figure 5C). It has been reported that the patients with long-term chronic pulmanary fibrosis may developed into lung cancer and the atypical adenomatous hyperplasia of lung is related with the development of adenocarcinoma of lung [9]. Of note, our case showed that the hyperplastic type alveolar epithelium had strongly positive expression of SP-A (Figure 5B), which is a major player in the pulmonary cytokine-network and to act in the pulmonary host defense [10], so further study is needed to detect the role of SP-A in the pathogenesis and prognostic evaluation of NSIP [11].

In summary, we described one case of INSIP from gross to light microscopy for removed lung. Three main findings were as followings: (1) The existences of heterogeneity and transmigration between these subtypes of INSIP, if it is the case, the predominant appearance should be considered for diagnosis. (2) The metaplasia of bronchiole epithelium and formation of cystic fibrous gas cavity are also pathological characteristic of INSIP-F rather than features only for UIP. (3) It is possible for small fibroblastic foci to appear in INSIP, which means INSIP might also have UIP-like regions. In a word, the CRP diagnosis is the best way for such puzzling cases. In addition, it is worth noticing that if patient has terminal interstitial lung disease and need the surgery of lung transplantation, it should pay attention to the selection of recipients and postoperative care**.**

## Consent

Written informed consent was obtained from the patient for publication of this case report and any accompanying images. A copy of the written consent is available for review by the Editor -in-chief of this journal.

## Abbreviations

INSIP: Idiopathic nonspecific interstitial pneumonia; UIP: Usual interstitial pneumonia; NSIP: Nonspecific interstitial pneumonia; IPF: Idiopathic pulmonary fibrosis; INSIP-F: INSIP fibrotic pattern; IIP: Idiopathic interstitial pneumonia; CRP: Clinico-radiologic-pathological; NSIP-C: NSIP cellular Type; NSIP-F: NSIP fibrotic pattern; ATS: American Thoracic Society.

Xia Fang and Benfang Luo are the Co-first author.

## Competing interests

The authors declare that they have no competing interests.

## Authors’ contributions

YXH, FX, LBF have made substantial contributions to conception and design; YXH, LF, ZY, LHP have acquired and analysed data; YXH, ZSX, ZXY, GLJ have written the manuscript and revised important intelligent content. All authors read and approved the final manuscript.

## Authors’ information

Professor YXH is a director of department of pathology who has expert field of differential diagnosis of interstitial pulmonary disease, Tongji Hospital, Tongji University School of Medicine. Professor LHP is a director of department of respiratory medicine, and expert on the field of interstitial pulmonary disease treatment, Shanghai Pulmonary Hospital, Tongji University School of Medicine.
